# Induction of active demethylation and 5hmC formation by 5-azacytidine is TET2 dependent and suggests new treatment strategies against hepatocellular carcinoma

**DOI:** 10.1186/s13148-015-0133-x

**Published:** 2015-09-11

**Authors:** Sahar Olsadat Sajadian, Sabrina Ehnert, Haghighat Vakilian, Eirini Koutsouraki, Georg Damm, Daniel Seehofer, Wolfgang Thasler, Steven Dooley, Hossein Baharvand, Bence Sipos, Andreas K. Nussler

**Affiliations:** Eberhard-Karls University Tübingen, BG Trauma Clinic, SWI, Schnarrenbergstraße 95, 72076 Tübingen, Germany; Department of Stem Cells and Developmental Biology at the Cell Science Research Center, Royan Institute for Stem Cell Biology and Technology, ACECR, Tehran, Iran; Centre for Clinical Brain Sciences, Chancellor’s Building 49 Little France Crescent, Edinburgh, UK; Department of General Surgery, Universitätsmedizin Berlin, Berlin, Germany; Department of General, Visceral, Transplantation, Vascular, and Thoracic Surgery, University of Munich, Campus Grosshadern, Munich, Germany; Section Molecular Hepatology, Department of Medicine II, Medical Faculty Mannheim, Heidelberg University, Heidelberg, Germany; Department of Pathology, Eberhard-Karls University Tübingen, Tübingen, Germany

**Keywords:** TET, 5-Hydroxymethylcytosine, DNA methylation, 5-Azacytidine, Cancer biomarker

## Abstract

**Background:**

Global deregulation of DNA methylation is one of the crucial causes of hepato cellular carcinoma (HCC). It has been reported that the anti-cancer drug 5-azacytidine (5-AZA) mediates the activation of tumor suppressor genes through passive demethylation by inhibiting DNMT1. Recent evidence suggests that active demethylation which is mediated by ten-eleven translocation (TET) proteins may also be an important step to control global methylation. However, there exists a controversial discussion in which TET proteins are involved in the demethylation process in HCC. Therefore, we firstly wanted to identify which of the TETs are involved in demethylation and later to study whether or not 5-AZA could trigger the TET-dependent active demethylation process in HCC. HCC cell lines (Huh-7, HLE, HLF), primary human hepatocytes (hHeps), and tissues from both healthy (55 patients) and HCC patients (55 patients) were included in this study; mRNA levels of isocitrate dehydrogenase (IDH1, 2) and TETs (TET1–3) were studied via qPCR and confirmed by Western blot. The expression of 5hmC/5mC was determined by immunohistochemistry in human HCC tissues and the corresponding adjacent healthy liver. HCC cell lines were stimulated with 5-AZA (0–20 μM) and viability (Resazurin conversion), toxicity (LDH release), proliferation (PCNA), and 5hmC/5mC distribution were assessed. In addition, knockdown experiments on TET proteins in HCC cell lines using short interference RNAs (siRNAs), in the presence and absence of 5-AZA, were performed.

**Results:**

Our data applying qPCR, immunofluorescence, and Western blotting clearly show that *TET2* and *TET3* but not TET1 were significantly decreased in HCC tissue and different HCC cell lines compared to non-tumor liver tissues and hHeps. In addition, we show here for the first time applying knockdown experiments that 5-AZA is able to trigger an active TET2-dependent demethylation process with concomitant significant changes in 5hmC/5mC in HCC cell lines and hHeps.

**Conclusions:**

Our data clearly show that the expression and activity of TET2 and TET3 proteins but not TET1 are impaired in hepatocellular carcinoma leading to the reduction of 5hmC in HCCs. Furthermore, this study identified a novel function of 5-azacytidine in promoting a TET-mediated generation of 5hmC suggesting that the availability of 5-AZA in cancer cells will have various effects on different epigenetic targets. These findings may open new therapeutic strategies for epigenetic drugs to treat HCC.

## Background

In the past years, epigenetic studies have focused on DNA methylation in mammals. DNA methylation plays an important role in genomic imprinting, especially in the inactivation pattern of X-chromosome [1, 2], the silencing of retrotransposon, and activation of tissue-specific genes. Recently, it has been demonstrated that these epigenetic changes have an influence in various pathologies and gene therapies, for instance, in cancer [3], viral infections [4], and the activity of mobile elements [5].

Methylation at the C-5 position of cytosine (C) has long been considered to be the only biologically functional epigenetic modification of the animal genomic DNA. In mammalians, 5-methylcytosines (5mC) established by DNA methyltransferases (DNMTs), mainly at CpG dinucleotides, is associated with gene repression [6–8].

Loss of methylation was observed during the development processes. Therefore, it was suggested that DNA methylation is reversible either through passive demethylation via DNA replication or by active demethylation via DNA repair followed by base excision repair of modified nucleotides or DNA glycosidase [9–11]. Moreover, it has been shown in many studies that active demethylation does occur in many mammalian cells [12]. The most well-known example is the rapid loss of 5mC immunoreactivity of the paternal pronucleus in the zygote after fertilization [13]. The active DNA demethylation pathway is enabled by the ten-eleven translocation proteins (TETs), named after the ten-eleven translocation (q22, q23) (10, 11) occurring in rare cases of acute myeloid and lymphocytic leukemia, which all belong to the family of Fe(II)/2-oxyglutarate-dependent dioxygenases [9, 14]. 5mC is oxidized by TET proteins and thereby converted to 5hmC, 5fC, and 5CaC [9, 15]. TET proteins mediate the DNA demethylation process and increase the diversity of the epigenetic state of genomic DNA. It was suggested that 5hmC represents an intermediate product in the active DNA demethylation process possibly following a demethylation mechanism through the thymidine salvage pathway to repair DNA alkylation damages [11].

The loss of 5hmC, together with a downregulation or mutation of TET proteins, have been reported in numerous types of cancer, e.g., melanoma, breast, prostate, myeloid, brain, and liver cancer [16, 12]. For example, mutations of TET2 protein have been recorded in patients with different myeloid malignancies [17–19]. A number of studies have provided an important link between TET2 dysfunction, an oncogenic metabolite 2-hydroxyglutarate (2-HG), myeloid differentiation, and malignancies [20, 21].

Hepatocellular carcinoma (HCC), which is frequently caused by a hepatitis virus B and C infection or alcohol abuse, is the most common type of primary liver cancer [22, 23]. The mechanisms that lead to HCC development are still unclear. Beside many possibilities that may lead to HCC development, it has been shown that accumulation of genetic abnormalities such as chromosomal alterations and gene amplifications, mutations along epigenetic alterations can trigger HCC development [24]. Recently, a particular focus has been directed towards abnormal DNA methylation at the fifth position of cytosine (5mC) [25]. Therefore, the detection of DNA methylation may provide a potential application for the clinical diagnosis of HCC. Liu and colleagues have reported that a reduction of 5hmC is associated with progression of liver cancer through downregulation of TET1 protein [18]. In contrast, Gao et al. have studied nine samples and reported not only the downregulation of *TET1* but also of *TET2* mRNA levels with a concomitant decrease of 5hmC. The researchers, however, found no change in *TET3* expression in hepatocellular carcinoma compared to normal liver samples [26]. Moreover, in another study by Yang et al., the decrease of all three *TET* genes was shown in three pairs of frozen human hepatocellular carcinoma tissue compared to matched normal liver tissue [27]. Despite accumulating evidence for the correlation between loss and decrease of 5hmC and progression of hepatocellular carcinoma, it remains totally unclear, which of the TET proteins seems to be responsible for the loss of active demethylation pattern in HCC.

In contrast to conventional or molecularly targeted therapies for inhibiting dysregulated genes or signaling pathways in HCC, epigenetic drugs may provide an alternative approach by reversing the methylation status. 5-Azacytidine is known as a DNA methyltranferase inhibitor (DNMTi), which is clinically approved for the treatment of myelodysplasia syndrome and acute myelogenous leukemia (AML) [28, 29]. However, the role of 5-azacytidine in active demethylation pathway is not clear.

Therefore, because of the apparent debate, which TET proteins are involved in 5hmC/5mC regulation in HCC, our primary aim of this study was to identify which TET protein play a crucial role in the regulation of 5hmC and 5mC in HCC. Furthermore, we wanted to know whether or not 5-AZA triggers a TET-dependent active demethylation process in HCC controlling 5hmC/5mC regulation.

## Methods

Cell culture medium, DMEM medium, William’s medium E, and cell culture supplements were purchased from Sigma-Aldrich (Steinheim, Germany). Cell culture plastics, phosphate buffered saline (PBS), and fetal calf serum (FCS) were purchased from PAA Laboratories GmbH (Pasching, Austria). DNaseI (RNase—free) and first strand cDNA Synthesis Kit were purchased from Fermantas (Ontario, Canada). 5-Azacytidine (SLBH7350V) was obtained from Sigma-Aldrich (Steinheim, Germany). All other chemical compounds were purchased from Carl Roth (Karlsruhe, Germany). 5hmC (39769) rabbit pAB and 5mC (39649) mouse mAB were purchased from Active Motif (Carlsbad, CA, USA). Proliferating cell nuclear antigen (PCNA) (ab92552) rabbit mAB was obtained from Abcam (Cambridge, UK). Corresponding secondary antibodies goat anti-rabbit Alexa 555 and goat anti-mouse 488 were acquired from Invitrogen (Carlsbad, CA, USA). Anti-TET2, anti-TET3, and anti-glyceraldehyde 3-phosphate dehydrogenase (GAPDH) antibodies were used from Sigma-Aldrich (Munich, Germany). The HRP-linked anti-rabbit IgG secondary antibody was purchased from Cell Signaling (Beverly, MA, USA).

### Tissue samples and primary human hepatocyte isolation and cell culture condition

Tissue specimens were obtained from patients undergoing resection of HCC according to the approval of local ethics committee. A tissue microarray (TMA) containing HCC samples and their corresponding noncancerous liver tissue was constructed. Primary human hepatocytes were isolated from human liver tissue according to the institutional guidelines of the Tubingen University from liver resections of tumor patients with primary or secondary liver tumor (ethics approval number: 368/2012BO2). The isolation and purification of primary human hepatocytes were performed as previously described [30]. Culture condition of HCC cell lines (Huh7, HLE and HLF) and human primary hepatocytes (hHeps) was published previously [31, 32, 30]. HLE and HLF cells were purchased from ATCC, and Huh7 was purchased from JCRB (Japanese Collection of Research Bioresources Cell Bank). The HCC cell lines as well as hHep were plated onto 6-, 24-, or 96-well plates, treated for 24 h and subsequently incubated with different concentrations of 5-AZA (20, 10, 5, 1) for 48 h. Huh7, HLE, and HLF cells were cultured in DMEM (Lonza Group Ltd., Cologne, Germany) supplemented with 2 mM glutamine (PAA Laboratories GmbH, Pasching, Austria) and 10 % FCS (Invitrogen, Darmstadt, Germany) at 37 °C and 5 % CO_2_ in a humidified incubator. Experiments were conducted between passage numbers 2 and 12 without full confluency throughout experiments. Absence of mycoplasma contamination was confirmed by Venor®GeMtest (Minerva Biolabs GmbH, Berlin, Germany).

### Immunohistochemical staining

Immunohistochemical stains were performed using 3.5 μm sections of TMA. Slides were deparaffinized, dehydrydrated, and after antigen retrieval were placed in 2 N HCL for 60 min, rinsed with distilled water, and placed in 100 Mm Tris–HCl (pH 8.5) for 10 min. Tissue slides were incubated in Dako Dual Endogenous Enzyme Block (CA, USA) for 10 min. The sections were blocked in PBS solution containing 10 % FCS and 0.1 % Tween-20 for 1 h. The section was incubated with either anti-5hmC rabbit at 1:1000 (active motif, 39769) or anti-5mC mouse at 1:500 (active motif, 39649) overnight at 4 °C and then incubated with Plus HRP One-Step Polymer kit from Zytochem (Berlin, Germany) for 30 min. Tissue sections were washed and subsequently DAB Substrate Kit High Contrast from Zytochem (Berlin, Germany) was used according to the manufacturer’s instruction. After rinsing, the cells were counterstained with hematoxylin. Scoring for immunohistochemical staining was performed by SAS and BS. All specimens were evaluated according to the 0–4 grading criteria (based on the percentage of 5hmC positive cells) and 0–3 grading criteria (based on the staining intensity) [33]. The intensity score was multiplied with percentage of positive cells of three different high power fields for each section. The total score was determined from 0 to 12.

### Immunofluorescence staining

Cells were plated onto cover slips and treated according to the experimental setup. Cells were fixed with 4 % paraformaldehyde solution for 15 min at RT and then washed with PBS. For permeabilization, cells were incubated with 0.5 % Triton-X-100 PBS solution for 15 min at RT. To denature the DNA, cells were incubated with 4 M HCl for 15 min at RT, rinsed with distilled water, and placed in 100 mM Tris–HCl (pH 8.5) for 10 min. After washing with PBS, unspecific binding sites were blocked with blocking buffer (10 % FCS, 0.1 % Tween-20 in PBS) for 1 h at RT. Then, cells were incubated with primary antibodies such as anti-5hmC rabbit polyclonal IgG (Active Motif, CA, USA), anti-5mC mouse monoclonal IgG, (Active Motif, CA, USA) or anti-PCNA Rabbit mAB (Abcam, Cambridge, UK) at 1:1000, 1:400, and 1:200, respectively, in PBS solution containing 1 % FCS and 0.1 % Tween-20 overnight at 4 °C. After washing with PBS, cells were incubated with secondary antibody solution (Alexa-Fluor antibodies, Invitrogen, NY, USA) diluted 1:400 in PBS solution containing 1 % FCS, 0.1 % Tween-20 for 1 h at RT. The nuclei were counterstained by incubation with Hoechst 33342 solution (2 μg/ml in PBS) for 10 min at RT. After a final washing step with PBS, the stained cells were mounted with mounting medium (Fluoromount G, Southern Biotech, NJ, USA). Images of the staining were taken with an EVOS fluorescence microscope (AMG, Life technologies, MA, USA) under standardized condition, processed, and analyzed with Image J 1.45s software (NIH, USA) [34].

### RNA extraction, cDNA synthesis, and quantification of gene expression

Total cellular RNA was isolated with Trifast (Peqlab, Erlangen, Germany); 2–3 μg of the total RNA was digested with DNase I in order to remove remaining genomic DNA, according to the manufacturer’s instructions (Fermentas International Inc., Ontario, Canada). Complementary DNA (cDNA) was synthesized by Revert Aid First Strand cDNA Synthesis Kit (Fermentas International Inc., Ontario, Canada).

For quantitative real-time PCR (qRT-PCR), 40 ng of template cDNA was used for the expression level of each target gene (primer sequences are listed in Table 1) using SYBR Green qPCR (Thermo scientific, Waltham, MA, USA) and the Step One Plus® Real-Time PCR System kit (Life technologies, Carlsbad, CA, USA). Among different existing housekeeping genes (e.g., *GAPDH*, *ACTB*, and *B2M*), *B2M* was used as an endogenous control because of the constant expression of this gene in hepatocellular carcinoma and normal tissue samples. In line with our results, Waxman et al. reported the dysregulation of common housekeeping genes such as *GAPDH* and *ACTB* in hepatocellular carcinoma [35], which may lead to wrong positive or wrong negative results. In preliminary experiments, we found that the expression of *TET1* was at the detection limit of all liver tissue samples and HCC cell lines. Therefore, we investigated isocitrate dehydrogenase (*IDH)1*, *IDH2*, *TET2*, and *TET3* and not *TET1* in our samples. Relative expression values were calculated from Ct values using the ΔΔC_T_ method with untreated cells as a control. PCRs were performed as follows: denaturation for 10 min at 95 °C, amplification with 40 cycles and 15 s at 95 °C, 40 s at 60 °C, and 15 s at 72 °C (StepOnePlus^TM^ Real-Time PCR System, Life technologies, CA, USA). The primer sequences are listed in Table 1.

**Table 1:** Sequences of primers used in qPCR

	Gene bank ID	Forward primer	Reverse primer	Product length (bp)
TET1	NM_030625.2	TCTGTTGTTGTGCCTCTGGA	GCCTTTAAAACTTTGGGCTTC	77
TET2	NM_001127208.2	GAGACGCTGAGGAAATACGG	TGGTGCCATAAGAGTGGACA	258
TET3	NM_001287491.1	CCCACAAGGACCAGCATAAC	CCATCTTGTACAGGGGGAGA	129
IDH1	NM-001282387.1	TCCGTCACTTGGTGTGTAGG	GGCTTGTGAGTGGATGGGTA	128
IDH2	NM-002168.3	TGAACTGCCAGATAATACGGG	CTGACAGCCCCCACCTC	121
GAPDH	NM_002046.4	TGCACCACCACTGCTTAGC	GGCATGGACTGTGGTCATGAG	87
B2M	NM_004048.2	AGATGAGTATGCCTGCCGTG	GCGGCATCTTCAAACCTCC	105

### Resazurin conversion, LDH leakage, and FACS measurement

To investigate the impact of 5-AZA on cell proliferation, the cells were incubated with different doses of 5-AZA at different time points. Cell viability (mitochondrial activity) was determined by resazurin conversion. Briefly, 1/10 volume of the resazurin stock solution (0.025 % in DPBS) was added to the cells. After 30 min of incubation at 37 °C, fluorescence was measured (Ex/Em = 544/590 nm) and corrected to background control (solvent mixture without cells). Viability is given as percentage of control (untreated cells).

LDH released into cell culture media as index of cell death was measured using an LDH assay kit from Analyticon (Lichtenfels, Germany) according to the manufacturer’s protocol. LDH released into the media was expressed as the percentage of total cellular LDH per well measured after cells had been lysed with 1 % Triton X-100.

Apoptosis was determined by fluorescence-activated cell sorting (FACS) quantification of hypodiploid cells. For FACS analysis of sub2N peaks, 1 × 10^6^ HCC cells per well were seeded in six-well plates and treated with different concentrations of 5-AZA. After 48 h, cells were fixed in ethanol and stained in hypotonic solution with RNase and Propidium Iodid (PI) for 30 min. Hypodiploid (sub2N) cells were considered apoptotic [36].

### Western blot

HCC cell lines and hHep cells were lysed in ice-cold RIPA lysis buffer (50 mM Tris, 250 mM NaCl, 2 % Nonidet-P40, 2.5 mM EDTA, 0.1 % SDS, 0.5 % DOC, complete protease inhibitor, 1.0 % phosphatase inhibitor, pH 7.2). Protein concentration was determined by micro-Lowry. Fifty micrograms of total protein was separated by SDS-PAGE and transferred to nitrocellulose membranes (Roth, Karlsruhe, Germany). The membranes were blocked by 5 % BSA solution for 2 h and incubated overnight with rabbit polyclonal primary antibodies (TET2, TET3, and GAPDH) (TET2, TET3 antibodies prepared 1:500 and GAPDH 1:10,000 in 5 % *w*/*v* BSA 1× TBS, 0.1 % Tween-20) (Sigma Aldrich, Munich, Germany) at 4 °C. The following day membranes were incubated with the corresponding HRP-labeled secondary antibodies for 2 h at RT. Chemiluminescent signals were detected with the ChemoCam (INTAS, Gottingen, Germany).

### Protein depletion by short interference RNAs

For depletion of *TET2* and *TET3,* the siRNAs were ordered from Eurofins MWG Operon (Ebersberg, Germany) and upon arrival tagged with fluorochrome. The short interference RNAs (siRNAs) were transfected with lipofectamine 3000 (Invitrogen, Carlsbad, CA, USA). The cells were seeded in six-well plates at the density of 2 × 10^5^ in 2 ml medium. siRNAs were transfected with lipofectamine reagent according to the manufacturer’s instructions. The final siRNAs concentration was 50 nM. Untreated cells and cells treated with 5-AZA were harvested after 48 h. The scrambled siRNA for negative control experiments were also obtained from Eurofins. The efficiency of transfection was measured by flow cytometry, and the efficiency of transfection was approximately around 70–80 % for each siRNAs in the HCC cell lines (data was not shown). The siRNA sequences are listed in Table 2.

**Table 2:** Oligonucleotide sequences for siRNA primers

Gene	Name	Target sequence	5′ or 3′ Mod
Control	Scrambled siRNA	5′AACAGTCGCGTTTGCGACTGG3′	3′ FLU
TET2	TET2-siRNA	5′CAAGACCAATGTCAGAA 3′	3′ FLU
TET3	TET3-siRNA	5′GATGAAGGTCCATATTA 3′	5′ CY3

### Statistical analysis

Statistical significance of differences between the individual treatments was evaluated by Student’s *t* test, one-way ANOVA, and whisker (Prism 5.01, GraphPad Software, San Diego, USA). Data are means ± SEM of three to five independent experiments. All statistical comparisons were performed two-sided in the sense of an exploratory data analysis using 0.05 (*), 0.01 (**), and 0.001 (***) level of significance.

## Results

### 5hmC is decreased in human HCC tissues and cell lines

**Fig. 1:**
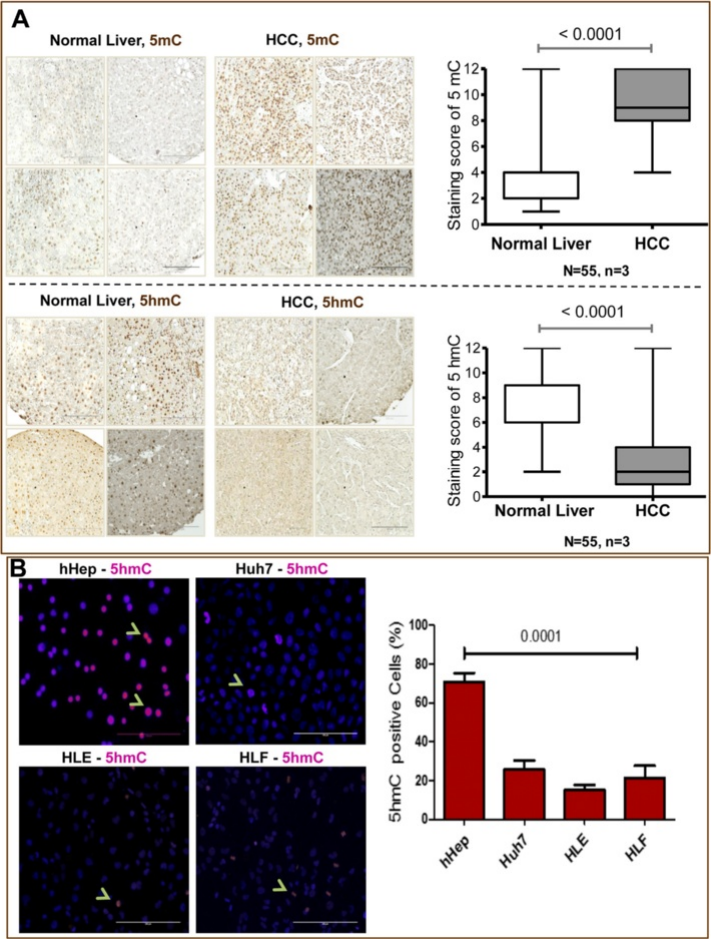
Distribution of 5-hydroxymethylcytosine (5hmC) and 5-methylcytosine (5mC) in HCC tissue. **a** IHC was performed using antibodies against 5hmC and 5mC in human normal and HCC tissues, the mean values of the IHC are shown (each group *N* = 55, *n* = 3). Four pictures are presented for each condition. The reduction of the 5hmC generation and an increase of 5mC in HCC samples compared with the corresponding normal liver samples were observed. Data are represented as box plot (whisker, Tukey). **b** IF staining was performed by using the antibody against 5hmC (*red*) for HCC cell lines (Huh7, HLE, and HLF). Cell nuclei were counterstained with Hoechst 33342 (*blue*). Scale bar are 20 μM. For quantification, 5hmC positive nuclei were counted. Statistical methods included paired *t* test and independent samples *t* test. All statistical tests were considered significant at *α* = 0.05 (*p* < 0.05)

The genomic distribution of 5hmC and 5mC in tissue-microarray-paraffin-embedded tissues and cells was evaluated by immunohistochemistry and immunofluorescence staining. When evaluating 55 HCC tissue samples, a significant (*p* < 0.001) decline of 5hmC (quantified via IHC, Fig. 1a) along with a significant (*p* < 0.001) increase of 5mC as compared to noncancerous liver tissue of the same patient (Fig. 1a) was observed in HCC liver samples. These results were confirmed in HCC cell lines showing a reduction of 5hmC as compared to primary human hepatocytes with immunofluorescence staining for 5hmC (Fig. 1b). These results are in line with previous reports demonstrating loss of 5hmC in various types of cancers, including HCCs [16, 18, 37].

### Reduction of 5hmC correlates with decreased TET2 and TET3 gene expression in HCC tissues and cell lines

**Fig. 2:**
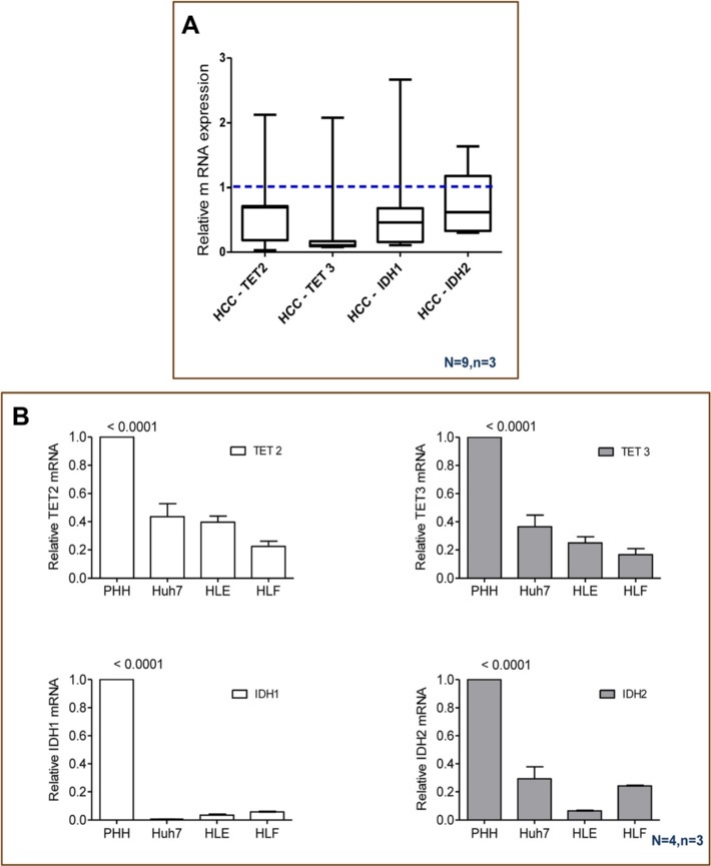
Reduction of 5hmC is associated with the substantial reduction of *IDH1* and *IDH2* and *TET2* and *TET3* gene expressions. **a** The expression of *TET2*, *TET3*, *IDH1*, and *IDH2* decreased in HCC tissues in comparison to the matched normal liver. Real-time qRT-PCR was used to determine *TETs* and *IDHs* relative mRNA levels in HCCs while using *β2M* as an internal control for normalization. Data are expressed as fold change in mRNA expression compared to the normal liver control (indicated by *dashed line* at *1*) The data are presented as box plot (*N* = 9, *n* = 3): center line represents the median, box limits represent the first and third quartiles, and whiskers represent 1.5 times the interquartile range (The *p* value were assessed by Student’s *t* test; bars show mean ± SE; *p* < 0.0001 for TET2, TET3, IDH1, and *p* = 0.0275 for IDH2). **b** The same pattern was confirmed by comparing HCC cell lines (Huh7, HLE, HLF) to primary human hepatocytes (*N* = 4, *n* = 3). (*p* value assed by one-way ANOVA; bars show mean ± SE) (*p* < 0.001)

To understand which important cellular factors are responsible for 5hmC reduction in HCCs, we determined the expression of *IDH1, IDH2*, *TET1, TET2*, and *TET3* by qRT-PCR. Despite others have reported that *TET1* is downregulated in HCC tissue compared with ‘normal’ liver tissue [18, 27], we found no *TET1* expression by qRT-PCR in normal adult human liver tissue, HCC tissue as well as HCC cell lines or human hepatocytes (data not shown). In contrast, we found that the expression of both *TET2* and *TET3* as well as the upstream genes *IDH1 and IDH2* were significantly decreased in human HCC tissues as compared to matched “normal” liver tissues (Fig. 2a). These data were confirmed in HCC cell lines (Huh7, HLE, and HLF) compared with freshly isolated primary human hepatocytes (Fig. 2b).

### Effect of 5-azacytidine on HCC cells proliferation and viability

**Fig. 3:**
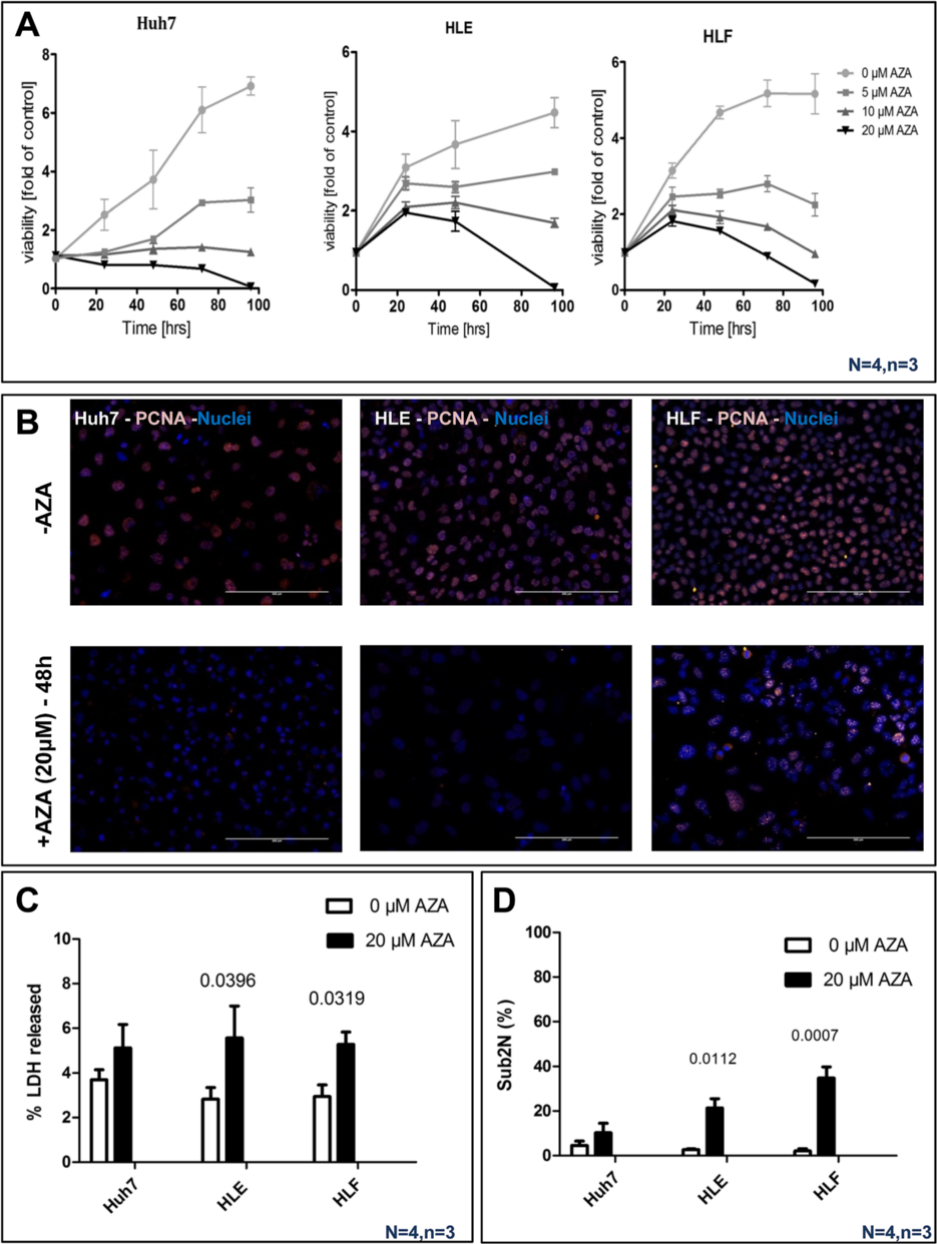
Distinct effect of 5-AZA on HCC cell lines viability, proliferation and apoptosis. **a** 5-AZA treatment reduced resazurin conversion in HCC cell lines (Huh7, HLE, and HLF). The mitochondria activity of cells decreased upon treatment with 5, 10, and 20 μM of 5-AZA for 24, 48, 96, and 120 h in time- and dose-dependent manner (*N* = 4, *n* = 3). **b** Twenty micromolars of 5-AZA inhibited the PCNA expression in HCC cell lines after 48 h of incubation. **c** Twenty micromolars of 5-AZA caused LDH release in HLE and HLF cells after 48 h; however, LDH released in culture supernants by Huh7 cells did not increase upon treatment 5-AZA after (*N* = 4, *n* = 3). **d** 5-AZA induced cell death in HLE and HLF cells. Determination of sub2N fractions as marker for apoptosis was measured after treatment of HCC cells with 20 μM 5-AZA for 48 h (*p* value assed by Student’s *t* test; bars show the mean ± SE of three independent experiments)

Incubation of the three tested HCC cell lines with 5-AZA showed a dose- and time-dependent reduction of mitochondrial activity, as measured by resazurin conversion (Fig. 3a). To strengthen this result, the effect of AZA on cell proliferation was additionally tested analyzing the proliferation cell nuclear antigen (PCNA) IF [38], showing a significant decrease nuclear staining compared to untreated cells (Fig. 3b). Altogether, our data show that incubation of HCC cells with 5-AZA inhibited proliferation in a dose-dependent manner in all tested HCC cell lines which is paralleled by an increased LDH release in HLF and HLE (Fig. 3c). Further analyses revealed that 5-AZA induced apoptosis to a certain extent in all tested HCC cell lines (Fig. 3d).

### 5-Azacytidine triggers active demethylation in HCC cells

**Fig. 4:**
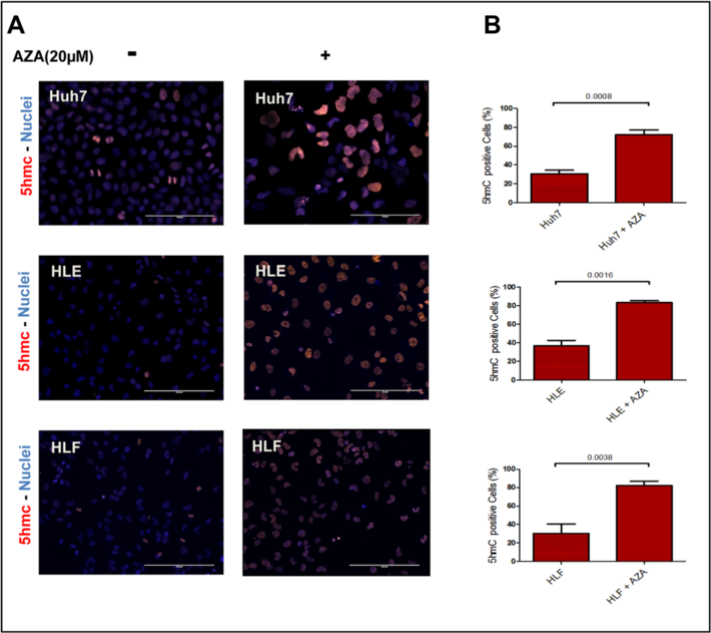
5-AZA enhanced the generation of 5hmC in HCC cell lines. **a** Immunostaining showed that 20 μM of 5-AZA enhances the generation of 5hmC in HCC cell lines after 48 h. **b** The semi-quantitative analysis of immunofluorescence staining indicated that the incubation of HCC cells with 5-AZA for 48 h increased 5hmC level (*p* value assessed by Student’s *t* test; data are represented as mean ± SE)

**Fig. 5:**
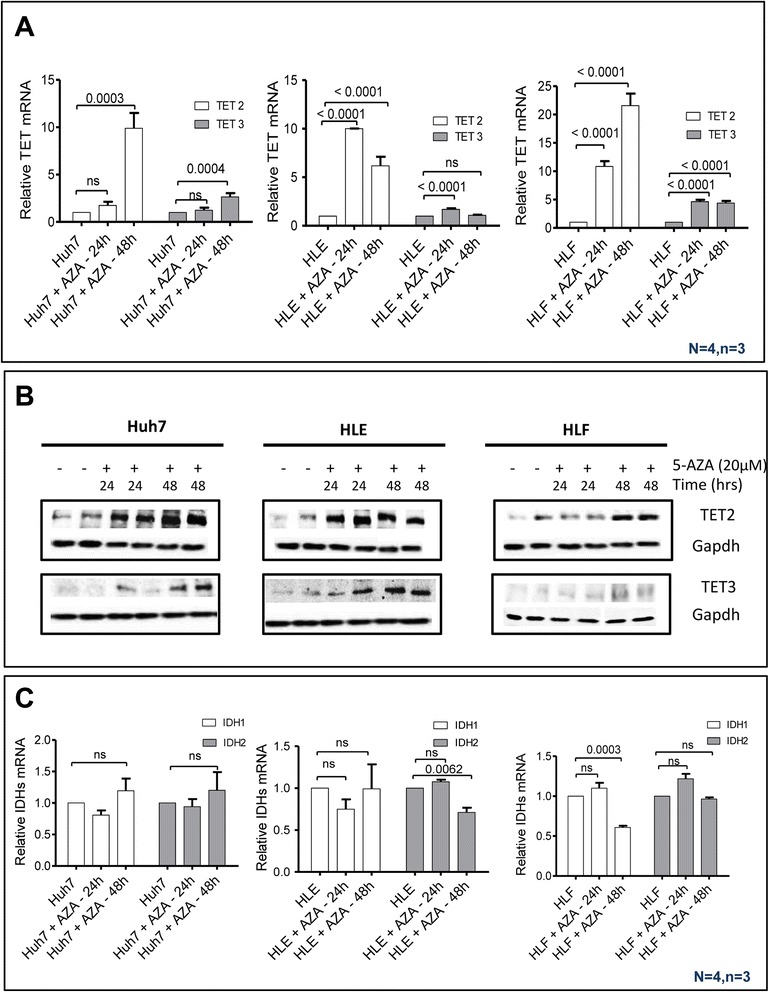
5-AZA enhanced the generation of 5hmC through induction of TETs expression in HCC cell lines. **a** Twenty micromolars of 5-AZA induced the expression of TET2 and TET3 in HCC cells after 48 h of incubation. The m-RNA level of *TET2* and *TET3* genes were determined by quantitative real-time PCR after 24 and 48 h (*p* value assed by one-way ANOVA; data represented as mean ± SE). **b** Expression of TET2 and TET3 was examined by Western blot analysis in HCC cell lines (Huh7, HLE, and HLF) after treatment with 20 μM 5-AZA for 24 and 48 h. **c** Twenty micromolars of 5-AZA did not influence the expression of IDH1 and IDH2 in HCC cells significantly after 48 h of incubation. Real-time qRT-PCR was used to determine *TETs* and *IDHs* relative mRNA levels in HCC cell lines while using *GAPDH* as an internal control for normalization

**Fig. 6:**
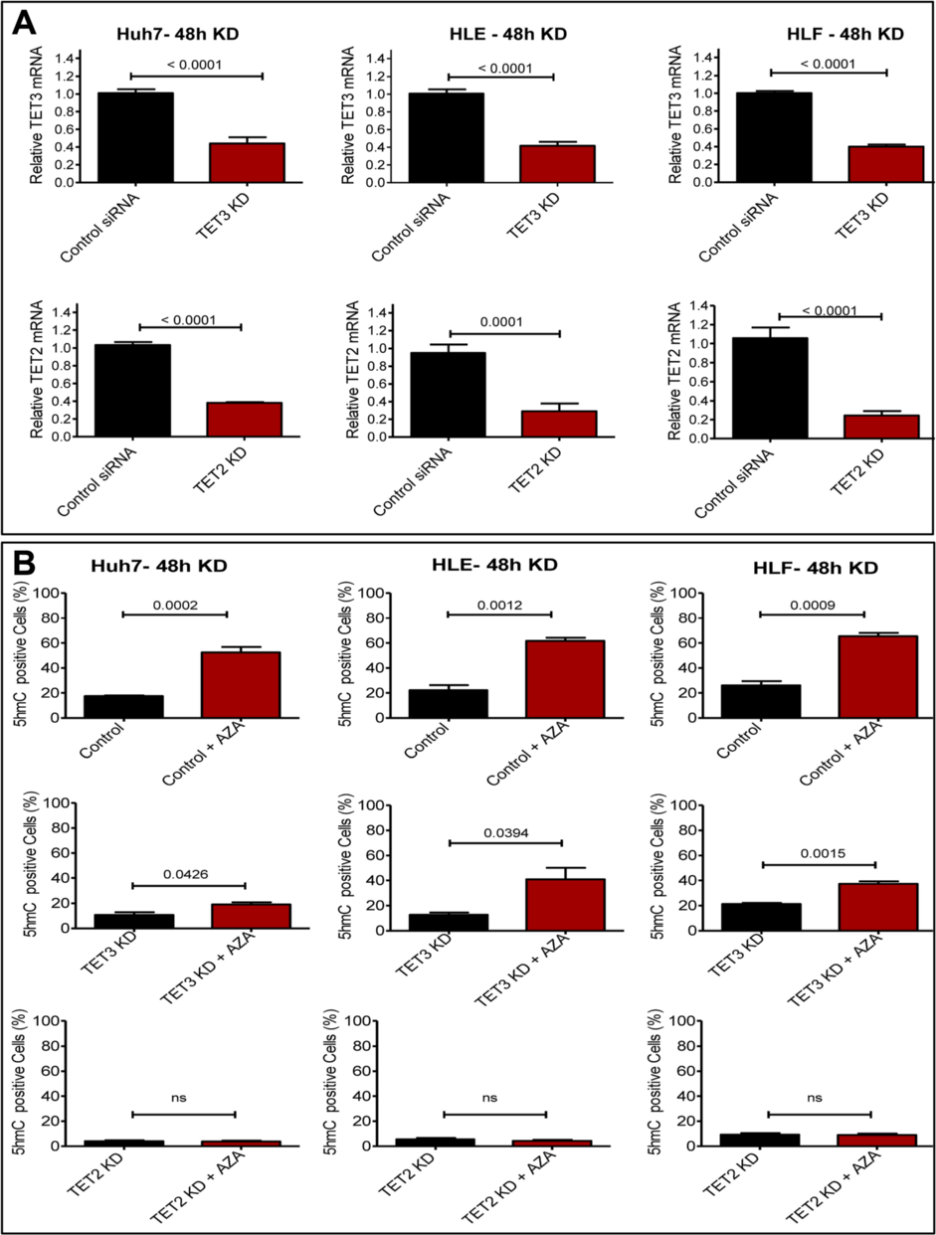
The effect of 5-AZA is mediated by TET methylcytosine dioxygenase. **a** siRNAs targeting *TET 2* and *TET3* decreased the level of *TET* m-RNA to approximately 30 % for *TET3* and 40 % for *TET2* as compared with corresponding cells transfected cells with control siRNAs shown by quantitative RT-PCR (*p* value assed by Student’s *t* test; data are represented as mean ± SE). **b** Immunostaining shows that the knockdown down of *TET* genes expression in HCC cell lines, specifically *TET2,* decreased the basal level of 5hmC signal and attenuated the induction of 5hmC by 5-AZA (20 μM) treatment for 48 h

It is known that 5-AZA induces passive demethylation through inhibition of DNMT [39]; however, it is not known to our knowledge if it has an influence on active demethylation through induction of *TET* expression. Treatment of HCC cell lines (Huh-7, HLE, and HLF) with 5-AZA for 24 and 48 h led to a significant increase in 5hmC-positive cells compared to untreated HCC cells (Fig. 4a). To further investigate a potential active demethylation process through converting 5mC to 5hmC, the expression of *TET2* and *TET3* was determined. 5-AZA-stimulated cells displayed significantly increased expression of *TET2* and *TET3*, while the expression of IDHs is not influenced (Fig. 5a, c). Increased expression of *TET2* and *TET*3 upon 5-AZA treatment was confirmed by Western blot analysis (Fig. 5b). We next needed to functionally prove if 5-AZA treatment in line with our previous work could trigger oxidation of 5mC to 5hmC through the induction of *TET2* and/or *TET3* expression [40] in HCC. If this would be the case, we would have strong evidence of an active demethylation process caused by 5-AZA. We were able to demonstrate that the conversion of 5mC to 5hmC upon 5-AZA treatment depends on TET proteins by simultaneous knockdown of *TET2* and *TET3* expression in Huh-7, HLE, and HLF cells, using siRNA. mRNA levels of the *TET* genes could be downregulated to approximately 30 % for *TET2* and 40 % for *TET*3, as compared to corresponding transfected cells with control siRNAs (Fig. 6a). In control cells, *TET2 and TET3* mRNA knockdown decreased intrinsic 5hmC levels. Upon *TET2* knockdown, 5-AZA (20 μM) treatment was unable to enhance the generation of 5hmC. In contrast, 5-AZA still enhanced—although to a much lesser extent—the expression of 5hmC in *TET3* knocked-down HCC cells (Fig. 6b). Overall, these results suggest that the enhancing effect of 5-AZA on 5hmC generation in HCC is mainly mediated by *TET2*.

## Discussion

Changing the balance of DNA methylation and demethylation related to epigenetic modification has become a main interest of cancer research in recent years [41]. The loss of 5hmC as a demethylation marker has been suggested as prognostic marker in various types of cancer and even to play a crucial role in the pathogenesis of cancers [42–44]. It was reported that 5hmC, in addition to its role in DNA demethylation via TET proteins, has a role in the regulation of genes involved in development, pluripotency, and the regulation of RNA splicing processes [45, 40]. It was shown that 5hmC is a major epigenetic modification mark in an adult human liver, playing an important role in hepatic gene expression changes in hepatocytes [46, 47]. In our study, we demonstrated that the generation of 5hmC is significantly reduced in hepatocellular carcinoma tissues and cell lines compared with healthy liver tissue and human hepatocytes. In line with other papers, our data clearly support the link between 5hmC ablation and tumor development [48, 49]. Furthermore, our study indicates that 5mC levels are induced in HCC tissues as well as cell in HCC cell lines. Altogether, our study confirms that detection of 5hmC could be a crucial diagnostic marker for HCCs.

TET proteins (TET1, TET2, and TET3) mediate the oxidation of 5mC to 5hmC. TET proteins were identified as dioxygenases that utilize two substantial factors: Fe(II) and 2-oxyglutarate (2-OG), in order to oxidize the methyl group (CH_3_) of 5mC, and to form 5hmC, thereby mediating active DNA demethylation [9, 14, 28, 50]. The expression of these three *TET*s varies not only in different organs but also in the development of organs [4, 14]. In various tumor types, a different *TET* family member has been reported to function as tumor suppressor [5–8]. Thereby, identification of the most effective TET protein leading to cancer in the corresponding tissue is a crucial topic to be investigated in different tumors. Recent evidence suggests that there is a remarkable correlation between decreased 5hmC levels and *TET* expression, resulting in progression of tumor and metastasis, suggesting that TET proteins might serve as a tumor suppressor in certain cancer types [37, 51]. Recently, it was reported that the gene encoding *TET2* but not *TET1* and *TET3* was frequently mutated, and it was identified as the relevant tumor suppressor gene, which is mutated in leukemia [17]. However, in breast cancer, low expression of *TET1* correlated with advanced cancer stage [52]. Furthermore, in some cancer types like colorectal cancer (CRC), the reductions of all three *TET* proteins have been reported [11, 53]. In addition, it was shown that *IDH1* or *IDH2* mutations could also result in the reduction of 5hmC in cancers [16, 33, 54]. In HCCs, however, the published results are contradicting each other regarding the role of *TET*s. Liu et al. reported that a reduction of 5hmC is associated with the downregulation of the TET1 protein in HCC [18] while Gao et al. claimed a downregulation of *TET1* and *TET2* but no change in *TET3* gene expression with a concomitant decrease of 5hmC in HCC [26]. Moreover, Yang et al. reported the decrease of all three *TET* genes in HCC; however, this group investigated only three pairs of frozen human hepatocellular carcinoma tissue compared to matched normal liver tissue [27]. In our study, we clearly found that only *TET2 and TET3* genes are significantly reduced in HCC tissue and in hepatocellular carcinoma cell lines. These changes were paralleled by a concomitant decrease of IDH genes and 5hmC and a significant increase of 5mC. The change in 5hmC and 5mC generation is confirmed by all papers [18, 26, 27], including our own results. But what may be the reasons for these conflicting results? Recently, it was shown that *TET1* plays a crucial role in the early development process in self-renewal pattern in embryonic stem cells [4, 14] suggesting a more crucial role for *TET1* in embryogenesis. Another hypothesis explaining the differences may be the infiltration of the so-called cancer stem cells with an embryonic pattern, which might be responsible for differences seen in *TET1* expression [55]. Another explanation could be simply the use of specific analytical tools. It has been reported that the expression of typical housekeeping genes including glyceraldehyde-3-phosphate dehydrogenase (*GAPDH*) and beta-actin (*ACTB*) can be affected through HCC development, and these housekeeping genes are expressed significantly different in HCC compared to normal liver tissue [35]. The use of instable genes for normalization may lead to an over- or underestimation of the fold changes or to misinterpretation of the results [35].

It is well established that 5-AZA causes the reduction of the DNA methylation status by inhibiting DNMT through passive demethylation pathway [56]. Recently, the active demethylation of DNA through oxidation of 5mC to 5hmC mediated by TET proteins was discovered [9]. To our knowledge, our study is the first to demonstrate that 5-AZA reduces the methylation status of DNA not only by triggering the passive demethylation pathway of DNA [56] but also the active demethylation pathway through converting 5mC to 5hmC by inducing *TET2* and *TET3* proteins. However, our results showed that 5-AZA was unable to induce the 5hmC in *TET2* knocked-down HCC cell lines suggesting a crucial role for *TET2* in 5hmC induction and in the pathogenesis of HCC. This was further underlined by our findings that 5-AZA stopped HCC tumor cell growth through decline of 5mC and a strong increase of 5hmC. However, a detailed mechanism of how 5-AZA affects the expression and activity of TET proteins remains to be further elucidated.

## Conclusions

The data presented here indicate that a drop of *TET2* and *TET3* expression and activity as well as *IDHs* is impaired in hepatocellular carcinoma with a concomitant reduction of 5hmC. Therefore, epigenetic drugs that mediate the conversion of 5mC to 5hmC through modulating *TETs* and/or *IDHs* activity may find broad use in the treatment of HCCs.

This study identified a novel function of 5-azacytidine in promoting a TET-mediated generation of 5hmC suggesting that the availability of 5-AZA in cancer cells will have various effects on different epigenetic targets.

## Abbreviation

5-AZA: 5-azacytidine
5caC: 5 carboxylcytosine
5fC: 5 formylcytosine
5hmC: 5 hydoxymethylcytosine
5mC: 5 methylcytosine
CRC: colorectal cancer
h: hours
HCC: hepato cellular carcinoma
hHep: human primary hepatocyte
IDH: isocitrate dehydrogenase
IF: immunofluorescence
IHC: immunohistochemistry
PCNA: proliferating cell nuclear antigen
siRNA: short interference RNA
TET: ten-eleven translocation
